# Lipoma of the Thumb: Spindle Cell Subtype

**DOI:** 10.1155/2016/9537175

**Published:** 2016-03-21

**Authors:** Johnny El Rayes, Roula Bou Sader, Elie Saliba

**Affiliations:** ^1^Orthopedic Surgery Department, “Hôtel-Dieu de France” Hospital, Beirut, Lebanon; ^2^Department of Radiology, “Bellevue” Medical Center, Beirut, Lebanon; ^3^Orthopedic Surgery Department, “Bellevue” Medical Center, Beirut, Lebanon

## Abstract

We report hereby the case of a 61-year-old man who presented with a soft-tissue swelling on the palmar aspect of the thumb. A detailed clinical examination followed by ultrasonography and excisional biopsy confirmed a spindle cell lipoma. Lipomas are rare in the hand and exceptional in the fingers, and we report, to our knowledge, the first spindle cell lipoma in the thumb to help in the differential diagnosis of a similar swelling.

## 1. Case Report

**Figure 1:**
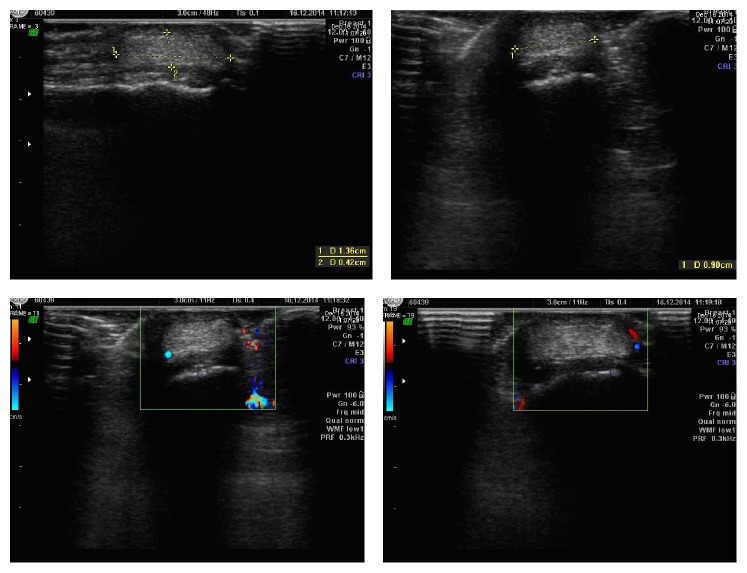
Ultrasound of the ulnar aspect of the thumb showing a well defined mildly hyperechoic lesion measuring 1.36*∗*0.42*∗*0.9 cm abutting the ulnar collateral ligament, which is otherwise intact. No internal flow was seen on Doppler.

A 61-year-old man presented with a swelling on the palmar aspect of the right thumb, at the level of the interphalangeal joint. The patient had noticed the emergence of a soft swelling after sustaining a benign trauma 3 months earlier. The lesion progressively increased in size to reach 2 cm of diameter. The patient experienced pain on movement. On clinical exam, the mass was soft, freely mobile, and painless on palpation. The overlying skin was normal. There was no sign of vascular or neurologic compression. Evaluation of the swelling by ultrasound showed a 1.4 cm well defined homogeneous and mildly hyperechoic lesion abutting the ulnar collateral ligament of the interphalangeal joint. No internal flow was detected on Doppler ultrasound (Figure 1). Differential diagnosis based on imaging characteristics included, amongst others, giant cell tumor of tendon sheath, nerve sheath tumor, and granuloma. Resection of the mass was performed at the operation room under local anesthesia and light sedation. A medial approach of the thumb was performed. We identified a white nodular encapsulated lesion of 1.5 cm that was in contact with the medial collateral nerve. It was easily dissected from the nerve and the surrounding soft tissues. The patient had an uneventful postoperative course, particularly with no paresthesia of the medial aspect of the thumb. Microscopic examination revealed a spindle cell lipoma, with no evidence of malignancy.

The patient was followed up for 2 years with no recurrence.

## 2. Discussion

Lipoma is by far the most common soft-tissue tumor, nearly accounting for 50% of all soft-tissue tumors [1] and approximately 16% of soft-tissue mesenchymal tumors [2]. Most common locations are in the upper back, neck, shoulder, and abdomen [3]. However, they rarely present in the hand with a reported incidence of 1% [4], and they often arise in the thenar or hypothenar regions [3]. Lipomas occur most commonly in the fifth and sixth decade [5] and are more frequently encountered in women [6]. De La Cruz Monroy in her literature review identified 36 cases of digit lipomas since Stein published the first lipoma of the finger in 1959 [3, 7]. The average age was 49 years. Both hands were affected equally. The index and middle fingers were most commonly involved (27 cases). Local invasion depended on the size. Moreover, in terms of location, only few cases affected the thumb and all these cases have presented with pain or restricted motion as did our patient [4, 7–9]. We noted that all patients were males: two were infants (8 and 9 years old) and two were adults (53 and 70 years old).

According to World Health Organization, lipomas are categorized into 9 entities, one of which is spindle cell/pleomorphic lipoma [2]. However, no case of spindle cell lipoma of the thumb adherent to the digital nerve has been reported in the literature. Ciloglu et al. presented a rare case of spindle cell lipoma adherent to the digital nerve in the palm [10]. This subtype of lipoma is a mixture of spindle cells, adipocytes, and collagen bundles and presents with an infiltrative pattern. Knowing that it can be misdiagnosed with a liposarcoma, it is important to know that it can appear in that location.

Clinically, most lipomas are asymptomatic [5]. Patients are often uncomfortable from limitation of mobility once they reach a significant size [2, 5]. Pain is not uncommon and it is usually mild [4]. Our patient in fact presented with both symptoms of pain and flexion limitation (approximately 20° of IP flexion).

Etiology and pathogenesis are still unclear. Multiple causative factors have been proposed including genetic, traumatic, and metabolic triggers [2]. Aust et al. reviewed 170 lipomas, of which 34 were qualified as “posttraumatic” [11]. They were all subcutaneous and superficial to the muscular fascia. In fact, lipomas may be superficial arising from the subcutaneous fat or less commonly subfascial, where they can have a larger size [5, 6, 12].

Differential diagnosis for a swelling in the hand is a large spectrum including neoplastic and nonneoplastic lesions [12]. However, the most common lesions are ganglion cyst, inclusion cyst, and giant cell tumor of tendon sheath [2, 3].

Imaging studies (CT scan and MRI) appear to be diagnostic in 71% of the cases [2, 4]. In our case, ultrasound imaging could not give a specific diagnosis. MRI would have surely been a better option to support the diagnosis of lipoma. In fact, in their retrospective study on 62 subjects with tumor-like finger lesions, Horcajadas et al. found that MRI can provide a highly accurate diagnosis in more than 80% of the cases [13].

Treatment depends mainly on the degree of functional impairment as well as on the cosmetic appearance [2]. Sarcomatous degeneration has not been described [1]. Surgical resection is the gold standard and requires extensive dissection to prevent recurrence. In our case, the patient complained of hand grips disturbance as well as cosmetic dissatisfaction.

The postresection recurrence rate of these subcutaneous lipomas is found to be less than 5% [1, 4]. With an adequate marginal resection, recurrence is rare (below 1%) [6].

## 3. Conclusion

To date, there are 4 cases of lipomas of the thumb reported. Our case adds to the previous ones and reminds us to always consider a benign lipoma in our differential diagnosis in swelling of the thumb, even though it is a rare entity.
